# Team emergency assessment measure (TEAM) of non-technical skills: The Brazilian Portuguese version of the TEAM tool

**DOI:** 10.1016/j.clinsp.2022.100043

**Published:** 2022-05-04

**Authors:** Fernando Rabioglio Giugni, Roger Daglius Dias, Caio Godoy Rodrigues, Henrique Trombini Pinesi, Augusto Scalabrini-Neto

**Affiliations:** a Pathology Department, Hospital das Clínicas, Faculdade de Medicina, Universidade de São Paulo (HCFMUSP), São Paulo, SP, Brazil; b Department of Emergency Medicine, Harvard Medical School, Boston, MA, USA; c STRATUS Center for Medical Simulation, Brigham and Women's Hospital, Harvard Medical School, Boston, USA; d Discipline of Clinical Emergencies, Hospital das Clínicas, Faculdade de Medicina, Universidade de São Paulo (HCFMUSP), São Paulo, SP, Brazil; e Heart Institute, Hospital das Clínicas, Faculdade de Medicina, Universidade de São Paulo (HCFMUSP), São Paulo, SP, Brazil; f Discipline of Clinical Emergencies, Hospital das Clínicas, Faculdade de Medicina, Universidade de São Paulo (HCFMUSP), São Paulo, SP, Brazil; g Abilities and Simulation Laboratory, Hospital das Clínicas, Faculdade de Medicina, Universidade de São Paulo (HCFMUSP), São Paulo, SP, Brazil

**Keywords:** Teamwork, Leadership, Communication, Education, Simulation

## Abstract

**Objectives:**

The aim of this study was to conduct the translation and cross-cultural adaptation of the original Team Emergency Assessment Measure (TEAM) tool into the Brazilian Portuguese language and investigate the internal consistency, inter-rater reliability, and concurrent validity of this new version (bp-TEAM).

**Methods:**

Independent medical translators performed forward and backward translations of the TEAM tool between English and Portuguese, creating the bp-TEAM. The authors selected 23 videos from final-year medical students during in-situ emergency simulations. Three independent raters assessed all the videos using the bp-TEAM and provided a score for each of the 12 items of the tool. The authors assessed the internal consistency and the inter-rater reliability of the tool.

**Results:**

Raters assessed all 23 videos. Internal consistency was assessed among the 11 items of the bp-TEAM from one rater, yielding a Cronbach's alpha of 0.89. inter-item correlation analysis yielded a mean correlation coefficient rho of 0.46. Inter-rater reliability analysis among the three raters yielded an intraclass correlation coefficient of 0.86 (95% CI 0.83‒0.89), p < 0.001.

**Conclusion:**

The Brazilian Portuguese version of the TEAM tool presented acceptable psychometric properties, similar to the original English version.

## Highlights

• The authors translated and cross-culturally adapted the TEAM tool into Brazilian Portuguese.
• The Brazilian Portuguese TEAM version proved to be a consistent and reliable tool.

## Introduction

Non-Technical Skills (NTS) can be defined as the cognitive, social, and interpersonal skills that complement technical skills and contribute to safe and efficient task performance.^1^ In healthcare, these skills include task management, teamwork, situation awareness, decision making, leadership, and communication.^2^ In the past decades, there has been great emphasis in training NTS among Emergency Department (ED) providers, especially in regards to patient safety and crisis resource management.^3^ Poor NTS is associated with unsafe behaviors and an increased incidence of adverse events in the ED.^4^ NTS can be trained using high fidelity simulation, which is already widely incorporated into educational curricula in graduate, residency, and continued medical education.^3^^,^^5^^,^^6^

There are several tools used to assess the non-technical performance of teams during emergency care. They are usually divided into observational tools, in which external raters observe and assess the team's performance, and self-assessment tools, in which the team members evaluate themselves.^7^ Both types have strengths and weaknesses, but observational tools have been more widely used and validated. Most observational NTS assessment tools use multiple-point scales to rate a set of NTS of the whole team. They usually differ in which skills are assessed, whether the scales reflect the frequency or quality of a certain skill, and in whether they can be used only in cardiac arrest or in general emergency situations. Among the many available observational instruments, the Team Emergency Assessment Measure (TEAM) tool was originally validated among medical students during emergency simulation scenarios and, more recently in multiple real and simulated ED settings with a variety of health professionals.^8^^,^^9^ The TEAM tool is easy to use, has a simple scoring system based on the frequency of observations and high inter-rater reliability.^9^, ^10^, ^11^, ^12^ The tool is composed of 11 items distributed in 3 domains (Leadership, Teamwork and Task Management), scored from 0 to 4, and a global NTS performance score from 1 to 10.^9^

The TEAM tool was translated and is available online in several languages, but the only published validated versions are English (original) and French languages.^9^^,^^13^ Currently, there is no validated translation of the TEAM tool to Brazilian Portuguese. The aim of this study was to conduct the translation and cross-cultural adaptation of the original TEAM tool into the Brazilian Portuguese language and investigate the internal consistency, inter-rater reliability, and concurrent validity of this new version (bp-TEAM) in high-fidelity simulations among final-year medical students.

## Materials and methods

### Study design

This study followed a stepwise approach for language translation, cross-cultural adaptation, and gathering initial validity evidence to create the bp-TEAM instrument.

### Translation and cross-cultural adaptation

**Figure 1:**
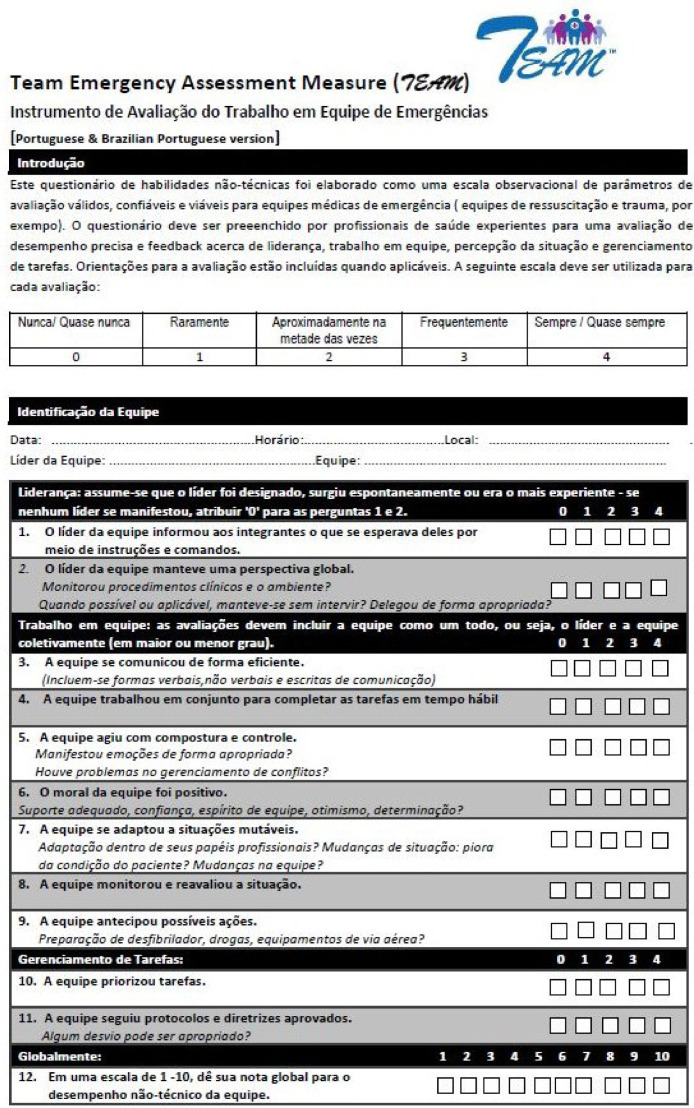
The Brazilian Portuguese TEAM tool (bp-TEAM).

Two independent medical translators translated the original TEAM instrument from English to Brazilian Portuguese (forward). The other three independent medical translators compared the translations and created a unique version by consensus. Then, this version was translated from Brazilian Portuguese to English (backward), and a unique English version was created by comparison and consensus. The author of the original TEAM received the backward translation version and provided comments and suggestions, which informed minor changes that were subsequently translated from English to Brazilian Portuguese, creating the final bp-TEAM version (Fig. 1).

### High-fidelity simulation scenarios

The authors selected a convenient sample of 23 videos from final-year medical students during simulated emergency situations in the Emergency Department (in-situ simulation) between 2014 and 2015. These simulations were part of their emergency medicine curriculum and were followed by debriefing, which was not recorded. Scenarios involved a team of 4‒6 students in one of the following major medical emergencies: septic shock, acute respiratory failure, acute coronary syndrome, and exogenous intoxication. The authors used the SIMMAN 3G human patient simulator and real medical equipment and supplies. Medications were replaced by saline.

### Raters

Three expert ED physicians were invited to observe and rate the simulation videos. The raters were trained on the bp-TEAM assessment by two experts during a 2-hour online session. All raters were native Brazilian Portuguese speakers.

### Procedures

Raters independently observed all 23 videos and rated the team's non-technical skills using an electronic version of the bp-TEAM instrument created in the *Qualtrics* platform. Raters were instructed not to pause, rewind or rewatch the same video. After observing each video, raters provided a score for each of the 11 items and the global score of the bp-TEAM tool.

### Statistical analysis

The authors described the score distribution as absolute numbers and percentages. Internal consistency was evaluated through the mean inter-item correlation coefficient (rho) among the 11 items calculated by the Spearman rank correlation test, and Cronbach's alpha coefficient. A correlation coefficient (rho) ≥ 0.4 and a Cronbachs' alpha ≥ 0.7 was considered satisfactory. Inter-Rater Reliability (IRR) was assessed with an Intraclass Correlation Coefficient (ICC) using a two-way mixed model. An ICC ≥ 0.7 was considered satisfactory. As conducted by the original TEAM validation study,^9^ in order to establish concurrent validity, correlation coefficients (rho) were calculated between each of the 11 items and the global performance score (Item 12). All analyses were performed using the software SPSS (version 20.4), and a p-value < 0.05 was considered statistically significant.

## Ethics

The study was approved by the institutional ethical committee of the *Hospital das Clínicas*, University of São Paulo Medical School (approval number #10245). All participants completed a written informed consent.

## Results

**Figure 2:**
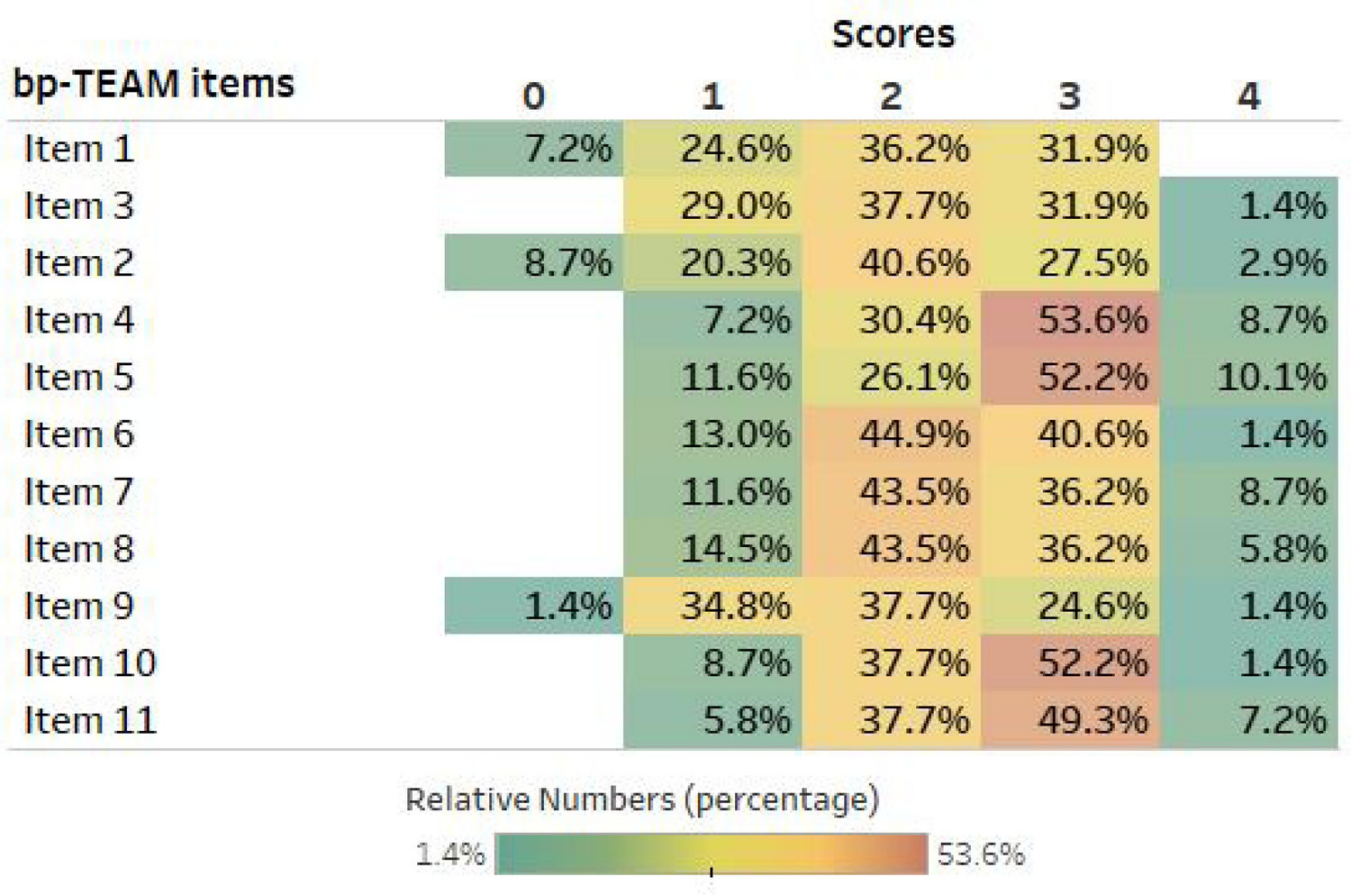
Distribution of scores used to rate 23 videos among all raters.

The three raters assessed all 23 videos, and none of the questionnaires and items had a missing value. The distribution (percentage) of the scores used by raters across all 11 items is shown in Fig. 2.

### Cross-cultural adaptation

Based on the backward translation, the author of the original TEAM made a total of seven minor corrections related to discrepancies on a few words between the translated and the original version (Table 1). All corrections were translated and integrated into the final bp-TEAM.

**Table 1:** Original TEAM author's comments.

Backward translation	TEAM author's comments
2 ‒ With some frequency	Not quite the same as "about as often as not" ‒ i.e., this measure is rating 50:50
Leadership: it is assumed that a leader was designated, if presented or was who had more experience ‒ if no leader expresses themselves, assign '0′ for questions 1 and 2.	Not quite right ‒ this is not quite what the original means
1. The team leader informed team members what was expected of them through guidance and commands.	Close ‒ but note that "guidance" does not have quite the same implication as "direction"
2. The team leader maintained a wide perspective. For example: supervised clinical procedures and the environment? Stayed away when necessary? Delegated appropriately?	Again close ‒ but "stayed away" is not quite the same as 'hands off'
11. The team followed patterns and approved guidelines: Can some deviation be approved?	Not quite the same ‒ "patterns" is not the same as "standards" and "can some deviation be approved" is not quite the same as "some deviation may be appropriate"
In general,	Not quite the same as "overall"
12. On a scale of 1‒10, give your general score/rating for non-technical team performance.	As above regarding "overall" as opposed to "in general"

Backward translated parts of the instrument with the original TEAM author's comments.

### Internal consistency

Internal consistency was assessed among the 11 items of the bp-TEAM from one rater, yielding a Cronbach's alpha of 0.89. Inter-item correlation analysis yielded a mean correlation coefficient rho of 0.46.

### Concurrent validity

Correlation analysis between each of the 11 items and the global performance score (Item 12) are summarized in Table 2. All items presented a moderate to strong correlation with the team's global performance (p < 0.05), except Item 9 (p = 0.221).

**Table 2:** Internal consistency.

Scale Items	Correlation Coefficient (rho)	p-value
Item 1	0.74	**< 0.001**
Item 2	0.83	**< 0.001**
Item 3	0.74	**< 0.001**
Item 4	0.63	**0.001**
Item 5	0.52	**0.010**
Item 6	0.59	**0.003**
Item 7	0.75	**< 0.001**
Item 8	0.59	**0.003**
Item 9	0.27	0.221
Item 10	0.82	**< 0.001**
Item 11	0.57	**0.005**

Correlation between individual items with the global performance score (n = 23).

### Inter-rater reliability (IRR)

IRR analysis among the three raters yielded an intraclass correlation coefficient of 0.86 (95% CI 0.83‒0.89), p < 0.001. Sub-analysis by domain showed the following: Leadership: 0.71 (95% CI 0.52‒0.83), p < 0.001; Teamwork: 0.42 (95% CI 0.25‒0.56), p < 0.001; Task Management: 0.61 (95% CI 0.36‒0.77), p < 0.001; Global Performance: 0.73 (95% CI 0.45‒0.88), p < 0.001.

## Discussion

The authors translated and conducted the cross-cultural adaptation of the original TEAM tool into the Brazilian Portuguese language. The bp-TEAM version proved to be a consistent and reliable tool, and these psychometric properties reflect important characteristics of high-quality assessment instruments.^14^

The bp-TEAM is the first NTS assessment tool in the Brazilian Portuguese language, validated to evaluate NTS in the emergency department. The authors followed the steps of existing guidelines of cross-cultural adaptation in the methodology.^15^ The psychometric properties of bp-TEAM, such as internal consistency and inter-rater reliability, are similar to the original English and the French versions.^9^^,^^13^ This study yielded a Cronbach alpha 0.89, comparable to the French version (0.95) and the original version (0.97). The mean intraclass correlation coefficient was 0.86, 0.93, and 0.60 in bp-TEAM, French TEAM, and original TEAM versions, respectively. Showing similar results to previous studies, especially the original TEAM version, highlight the psychometric robustness of the bp-TEAM tool.

This translated version of the TEAM tool has the potential to promote NTS education in Brazil. Simulation-based education is growing in Brazil, but NTS training is often conducted in an intuitive manner, using unstructured and informal assessment and feedback strategies. Having a proper assessment tool may help medical educators to evaluate NTS in a more systematic and objective way, which can help debriefing and feedback to be more effective. The Non-Technical Skills for Surgeons (NOTSS) behavior assessment tool is a fine example of an assessment tool that was later widely used as an educational tool for teaching and assessment of NTS in the surgical context.^16^, ^17^, ^18^ In fact, the NOTSS taxonomy is now used by the American College of Surgeons as an essential component of all surgical residency curricula in the US.^19^

There are a few limitations in the present study. It was research conducted in a single center and tested with only simulation videos of final-year medical students, which may limit its generalization among other groups of professionals or students. The raters were all physicians with the same background and training so the inter-rater reliability among other ED providers might not be the same. Bp-TEAM was designed and tested in a Brazilian Portuguese speaking environment, and it may need adjustments in other Portuguese-speaking countries with diverse cultural characteristics.

## Conclusion

This study developed a translation and cross-cultural adaptation of TEAM to Brazilian Portuguese with acceptable psychometric properties. This result is important for NTS training across Brazil and thus for emergency medicine in the country. Further studies are needed to show how the bpTEAM will impact medical education and clinical practice in Brazil.

## Authors’ contributions

Fernando Rabioglio Giugni: Data acquisition, manuscript first draft.

Roger Daglius Dias: Study design, data analysis and interpretation, manuscript draft and critical review.

Caio Godoy Rodrigues: Data acquisition, critical manuscript review.

Henrique Trombini Pinesi: Data acquisition, critical manuscript review.

Augusto Scalabrini-Neto: Study conception and supervision, data interpretation, manuscript critical review.

## Conflicts of interest

The authors declare no conflicts of interest.
